# CRISPR/Cas9-mediated mutagenesis of *ClBG1* decreased seed size and promoted seed germination in watermelon

**DOI:** 10.1038/s41438-021-00506-1

**Published:** 2021-04-01

**Authors:** Yanping Wang, Jinfang Wang, Shaogui Guo, Shouwei Tian, Jie Zhang, Yi Ren, Maoying Li, Guoyi Gong, Haiying Zhang, Yong Xu

**Affiliations:** National Watermelon and Melon Improvement Center, Beijing Academy of Agricultural and Forestry Sciences, Key Laboratory of Biology and Genetic Improvement of Horticultural Crops (North China), Beijing Key Laboratory of Vegetable Germplasm Improvement, Beijing, 100097 China

**Keywords:** Plant molecular biology, Seed development

## Abstract

Abscisic acid (ABA) is a critical regulator of seed development and germination. β-glucosidases (BGs) have been suggested to be contributors to increased ABA content because they catalyze the hydrolysis of ABA-glucose ester to release free ABA. However, whether BGs are involved in seed development is unclear. In this study, a candidate gene, *ClBG1*, in watermelon was selected for targeted mutagenesis via the CRISPR/Cas9 system. Seed size and weight were significantly reduced in the *Clbg1*-mutant watermelon lines, which was mainly attributed to decreased cell number resulting from decreased ABA levels. A transcriptome analysis showed that the expression of 1015 and 1429 unique genes was changed 10 and 18 days after pollination (DAP), respectively. Cytoskeleton- and cell cycle-related genes were enriched in the differentially expressed genes of wild type and *Clbg1-*mutant lines during seed development. Moreover, the expression of genes in the major signaling pathways of seed size control was also changed. In addition, seed germination was promoted in the *Clbg1*-mutant lines due to decreased ABA content. These results indicate that *ClBG1* may be critical for watermelon seed size regulation and germination mainly through the modulation of ABA content and thereby the transcriptional regulation of cytoskeleton-, cell cycle- and signaling-related genes. Our results lay a foundation for dissecting the molecular mechanisms of controlling watermelon seed size, a key agricultural trait of significant economic importance.

## Introduction

The seed is a unique organ in a seed plant and is crucial for the plant life cycle. Seed size and weight are important agronomic traits, and in addition to influencing plant fitness and adaption to environmental stresses, seeds can also affect yield and quality, which is especially true for plants for which seeds are the main product organ^1–3^. The seeds of watermelon (*Citrullus lanatus* (Thunb.) Matsum. & Nakai) have dual uses. Edible watermelon seeds provide humans with rich nutrition, such as oil and protein^4^; therefore, large seeds are preferred; in contrast, for flesh-consumed watermelons, no (or small and sparse) seeds are better because the flesh portion is larger, which improves consumer experience^1^. Therefore, seed size is an important horticultural trait for selection during watermelon breeding. However, the molecular mechanisms underlying seed phenotype remain unclear.

Seed development is a very complex process. Seed size regulation involves numerous genes that respond to developmental and environmental signals^5,6^. Recent studies have revealed some key genes and regulatory pathways that control seed size in plants^6,7^, including G protein signaling, the ubiquitin-proteasome pathway, mitogen-activated protein kinase (MAPK) signaling, some transcriptional regulators, and phytohormone perception and homeostasis. G protein signaling affects multiple aspects of physiological activities during plant growth and development. The functional G protein is a heterotrimeric complex composed of three subunits, namely, the Gα, Gβ, and Gγ subunits. In Arabidopsis, a Gγ (AGG3)-deficient mutant has small seeds^8,9^. In rice, GS3 and DEP1, which share significant similarity with Arabidopsis AGG3, were also found to be involved in grain size regulation^10,11^. Recent studies revealed that the regulation of these Gγs in grain length depends on Gα (RGA1), RGB1, and the transcription factor MADS1^12,13^. The ubiquitin-proteasome pathway has recently been revealed to be of importance in seed size regulation. In Arabidopsis, together with the E3 ubiquitin ligases DA2 and BB/EOD1, the ubiquitin receptor DA1 was found to regulate seed growth^14^. The homolog of DA1 in rice, wheat, and maize (GW2) was also reported to be involved in grain size determination^15–17^. Moreover, mutation in UBP15 (ubiquitin-specific protease 15) encoded by suppressor of DA1 (SOD2) led to decreased seed and organ size. In addition, mutations in factors participating with the 26S proteasome and anaphase-promoting complex/cyclosome (APC/C) ubiquitin ligases, such as SAMBA, cause large seeds^18^. Plant mitogen-activated protein kinase (MAPK) cascades, consisting of MAPK, MAPK kinase (MAPKK), and MAPKK kinase (MAPKKK), play important roles in a number of signal transduction pathways. In Arabidopsis, the *mkk4/mkk5* double mutant exhibited a short seed^19^. In rice, OsMKKK10, OsMKK4, and OsMAPK6 act tandemly to control grain size in a positive way^20^. In addition to the aforementioned factors, transcriptional regulators, including transcription factors, transcriptional coactivators, and chromatin modification regulators were also reported to participate in the regulation of seed size. The SQUAMOSA promoter-binding protein-like family of transcription factors, including members OsSPL13 and OsSPL16, positively affect rice grain size by modulating the expression of SRS5 and GL7, respectively^21,22^. OsmiR396, OsGRF4, and OsGIF cooperatively function to regulate rice grain size in a positive way^23^. The Arabidopsis B3 domain transcriptional repressor NGAL acts synergistically with KLU to regulate seed size^24^. In legumes, BS1, a plant-specific transcription regulator, was discovered to be critical for seed size determination^25^. Arabidopsis *SMOS1* and *TTG2*, encoding the AP2 and WRKY transcription factors, respectively, were also found to be important in the control of seed size^26^.

Plant hormones have diverse functions during plant growth and development. BR and auxin are considered important regulators of seed size^27^. BR biosynthesis and signaling genes affect seed size^28,29^. Moreover, auxin biosynthesis, transport, and signaling are critical for seed size control^30,31^. Another important hormone, abscisic acid (ABA), is known for its prominent role in seed dormancy and germination control^32^. However, its role in seed size regulation has rarely been reported. The biological function of ABA depends on both its levels and signal transduction. Endogenous ABA levels are upregulated by de novo biosynthesis and/or by the one-step hydrolysis of Glc-conjugated ABA (ABA-GE), as catalyzed by β-glucosidase (BG)^33^. In this study, using the CRISPR/Cas9 system, watermelon *Clbg1* mutants (mutations in *ClBG1*, whose partial coding sequence was reported by Li et al.^34^) were generated and found to cause decreased ABA content, smaller seed size and increased germination potential. To determine the mechanisms underlying these phenomena, paraffin sections were prepared to find an explanation at the cellular level. Moreover, we performed a transcriptome analysis at two relatively early seed development stages and two time points after seed imbibition in wild-type (WT) and *Clbg1*-mutant lines. The expression of genes in the major signaling pathways of seed size control was also investigated. The findings expand our knowledge of the role of ABA in seed size control and may be useful for breeding seed-edible watermelon.

## Results

### CRISPR/Cas9-engineered mutations in *ClBG1* led to decreased ABA content and seed size

**Fig. 1:**
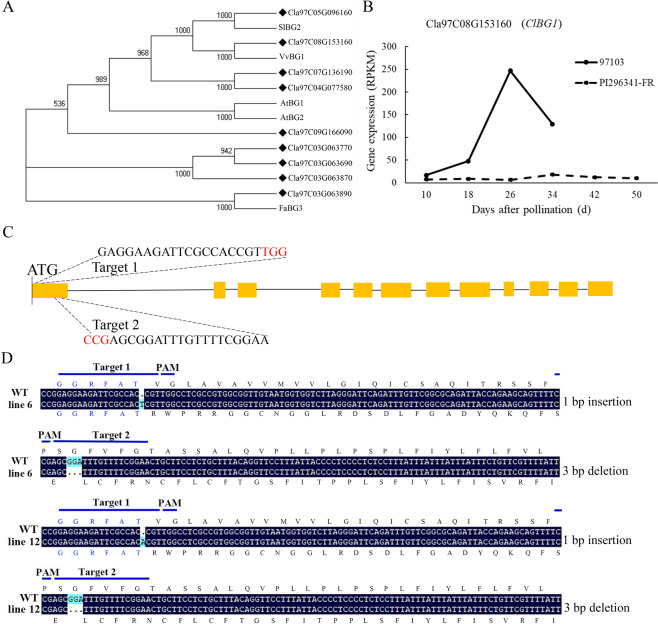
**A** Phylogenetic trees of the BG gene family. Genes from watermelon are labeled with black diamonds. The phylogenetic trees are shown only in the form of topology structures. Sequence data from other species can be found in the GenBank data libraries under the following accession numbers: tomato-SlBG2 (Solyc07 g063880), grape-VvBG1 (XM_002274626), Arabidopsis-AtBG1 (841670) and Arabidopsis-AtBG2 (824893), and strawberry-FaBG3 (JX244263). **B** Expression of *ClBG1* (Cla97C08G153160) in the 97103 and PI296341-FR lines during watermelon fruit development. **C** Schematic diagram of CRISPR/Cas9-targeted sites in the *ClBG1* gene. Orange boxes represent exons; lines represent introns. PAMs (NGGs) are shown in red, and the sequence of each gRNA is shown. **D** Mutation sites in the *Clbg1* homozygous mutant lines from the T4 generation. Frameshift mutations for encoding sequences are also shown

ABA is a critical factor in regulating watermelon fruit ripening^35^. In addition to NCEDs, which encode the rate-limiting enzyme in ABA biosynthesis, β-glucosidase also contributes to an increased level of free ABA. To isolate the putative BGs genes from the watermelon genome database, BLASTP searches of the watermelon protein database were performed based on the known BGs in tomato, grape, *Arabidopsis* and strawberry. Ultimately, nine *ClBG* genes were identified in watermelon, and three of these genes were negligibly expressed (Figs. 1A and S1). Among the six expressed *ClBG* genes in watermelon, the expression of *ClBG1* (Cla97C08G153160) in cultivated watermelon 97103 increased with fruit development, while it was relatively less expressed and more stable in wild watermelon PI296341-FR (Figs. 1B and S1), which is in accordance with ABA content variation during watermelon fruit development^35^, making it the top candidate for involvement in releasing ABA from its conjugate form in the process of watermelon fruit ripening.

To further investigate the function of *ClBG1* in watermelon, we mutated *ClBG1* through the CRISPR/Cas9 system in cultivated watermelon variety ZXJM. Two sequences located in the first exon were selected as targets (Fig. 1C), which were used to obtain the desired watermelon plants with dysfunctional *ClBG1*. In addition, to test whether there are potential off-target sites, the two target sequences of *ClBG1* were searched in the watermelon genome database (http://cucurbitgenomics.org/) by BLAST with a low *E*-value (1*e*^−1^). Except for the target sites, no additional sites were found in the watermelon genome, indicating that off-target events would be rare upon *ClBG1* mutation. As a result, 40 basta-resistant watermelon lines were obtained from the T0 generation, and Sanger sequencing analyses revealed that almost all these lines had the expected mutations at the targeted sites. To obtain homozygous *Clbg1* mutants without Cas9, a total of 60 T2 plants were detected, and two types of *ClBG1* mutations without Cas9 (first target, 1-bp insertion; second target, three-bp deletions) were obtained. These mutations in the first exon resulted in frame shifts (Fig. 1D).

**Fig. 2:**
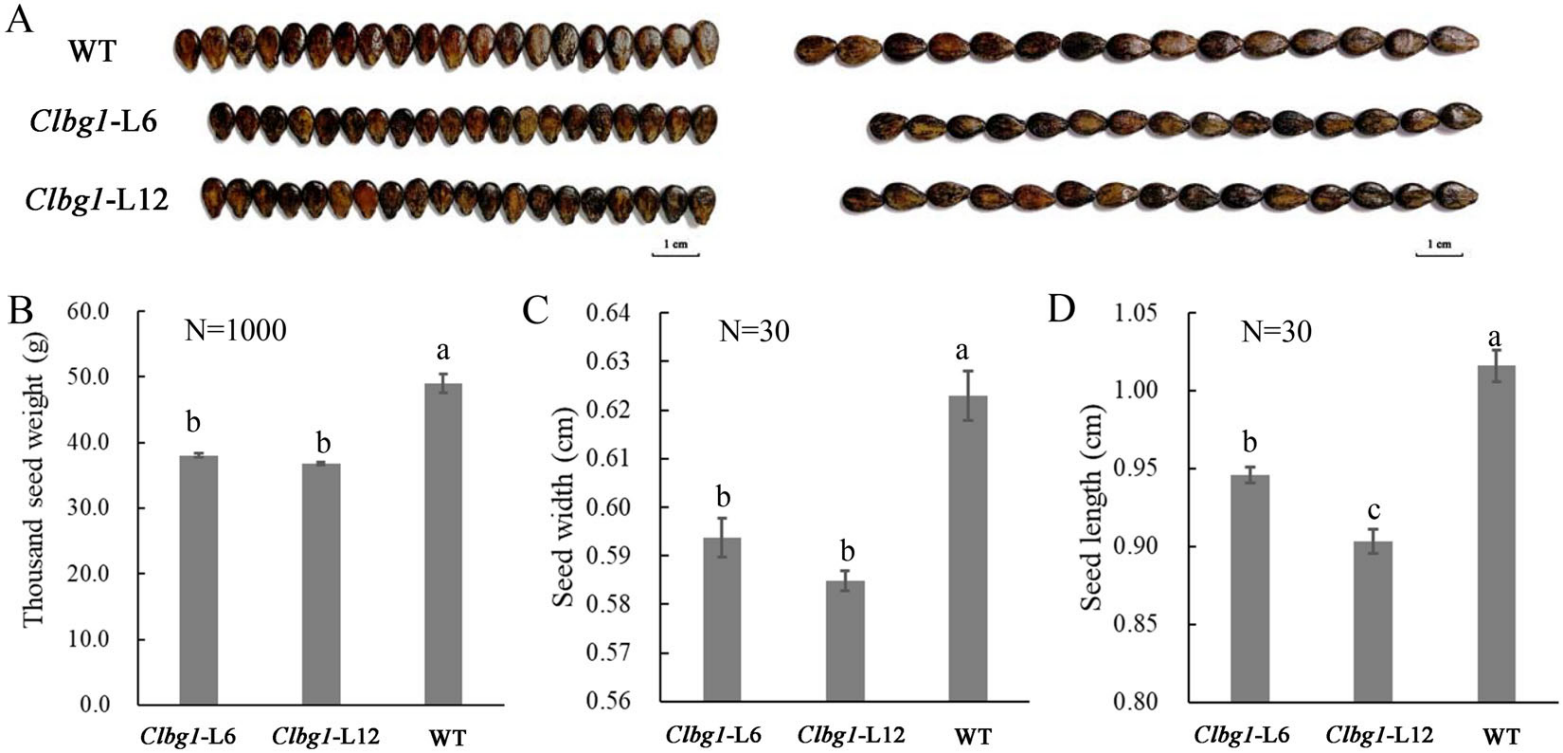
Seed size variation in the *Clbg1*-mutant lines. Seed phenotype (**A**); weight of one thousand seeds (**B**); average seed width (**C**) and length (**D**); N stands for the number of seeds that were analyzed for each genotype and each replication. Standard error is indicated. Different letters represent significant differences as determined using ANOVA followed by Tukey’s HSD test (*p* < 0.05)

**Fig. 3:**
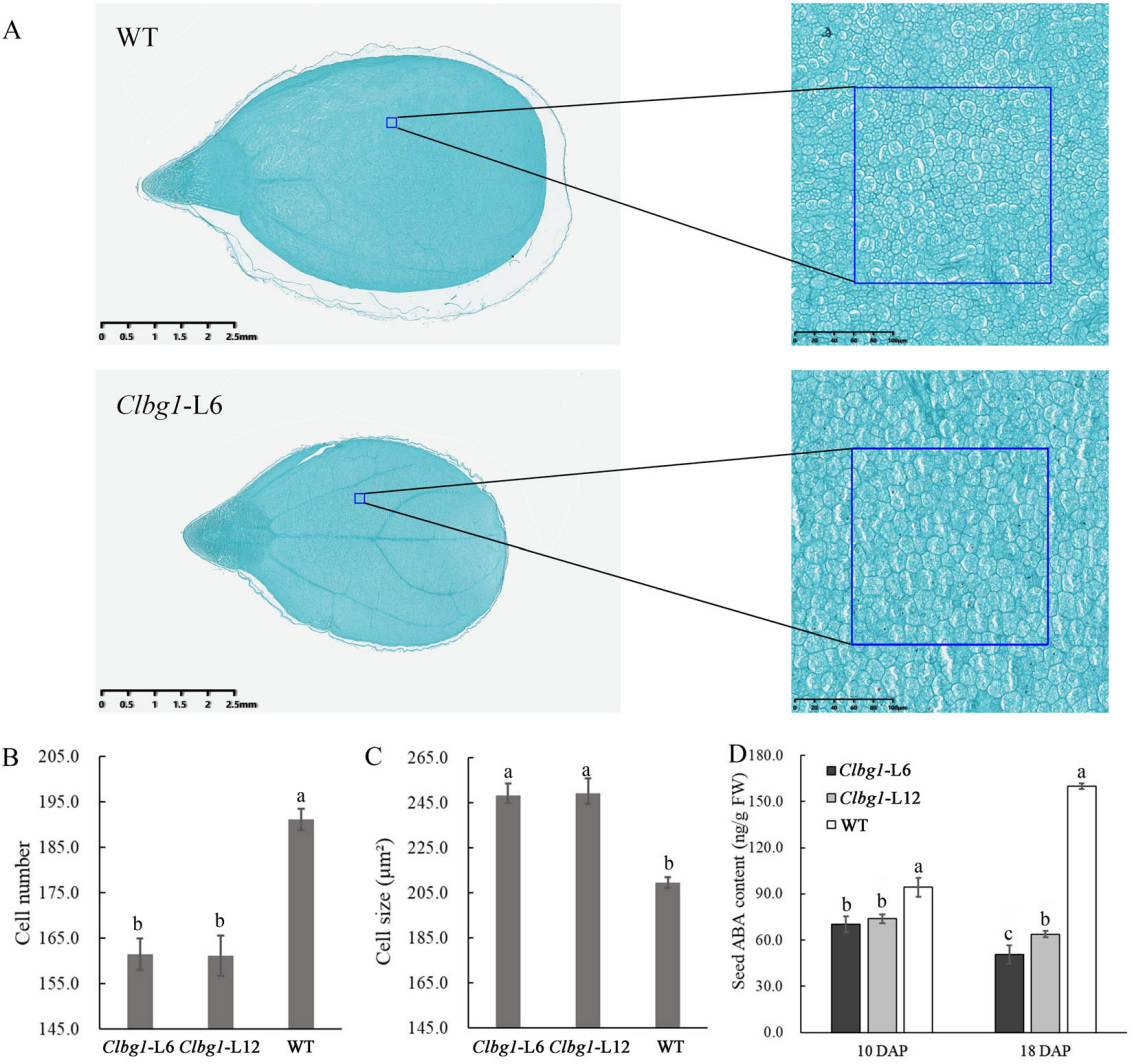
The *Clbg1* mutation affected seed size by decreasing cell number. Paraffin sections of mature WT and *Clbg1*-mutant seeds cut in the longitudinal direction (**A**); cell number (**B**) and cell size (**C**) in the mature seeds of the *Clbg1* mutants and WT plants; ABA content in the seeds 10 and 18 DAP (**D**); standard error is indicated. Different letters represent significant differences as determined using ANOVA followed by Tukey’s HSD test (*p* < 0.05)

As expected, the ABA content in *Clbg1*-mutant plant lines was significantly decreased (Fig. S2). Surprisingly, the process of fruit development and ripening was not significantly altered in the *Clbg1*-mutant lines compared to that in the WT line (Fig S3), while seed size was significantly decreased, including decreased seed weight, length, and width (Fig. 2). Therefore, more attention was given to the effect of *ClBG1* on watermelon seed development. To reveal the mechanisms of seed size variation in *Clbg1*-mutant lines at the cellular level, mature seeds were sectioned. The cell number was significantly decreased in the *Clbg1*-mutant lines compared to the WT line, while the cell size was significantly enlarged (Fig. 3A–C). In addition, the ABA content in seeds was detected. Our results showed that ABA was increased from 10 DAP to 18 DAP in the WT plants, which was consistent with our previous study^35^. However, in the *Clbg1*-mutant lines 6 and 12, the ABA content was lower at 18 DAP than at 10 DAP (Fig. 3D). As expected, the ABA content was significantly decreased in seeds of *Clbg1* mutants compared the WT plants at both 10 and 18 DAP (Fig. 3D).

### Cytoskeleton- and cell cycle-related genes were involved in watermelon seed size regulation

**Fig. 4:**
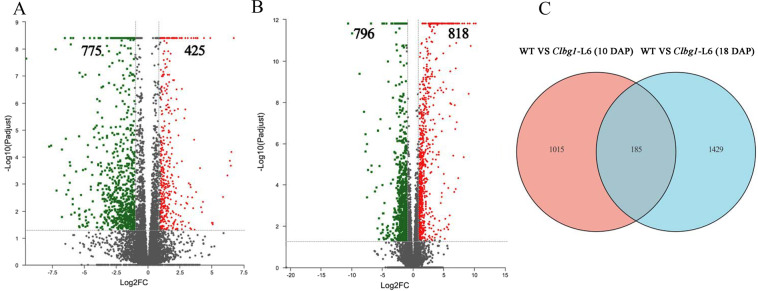
DEGs analysis 10 and 18 DAP. **A**, **B** Volcano plot showing differences in the WT and *Clbg1*-mutant lines 10 and 18 DAP, respectively. Green indicates the downregulated genes, and red represents the upregulated genes. **C** Venn diagram showing the number of specific and common DEGs 10 and 18 DAP

**Fig. 5:**
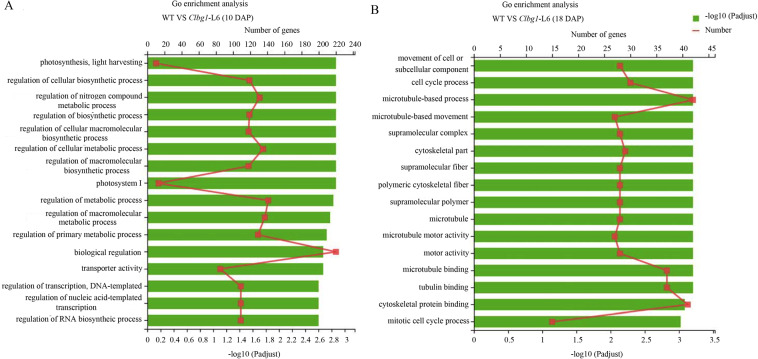
GO enrichment analysis of the DEGs between the WT and *Clbg1*-mutant lines 10 DAP (**A**) and 18 DAP (**B**). The green bars indicate the value of –log10 (padjust), and the lines indicate the number of genes. The threshold for DEGs was set at a fold change ≥2 and padjust < 0.05

To understand the reason(s) that seed size was decreased in the *Clbg1* mutants at the transcription level, a comparative transcriptome analysis of the wild type and *Clbg1*-mutant line was performed during two key seed developmental stages. Ten days after pollination (DAP), 1200 differentially expressed genes (DEGs) were identified, with 775 downregulated and 445 upregulated in the *Clbg1*-mutant lines (Fig. 4A). Moreover, 18 DAP, 1614 genes were significantly changed in the *Clbg1*-mutant lines, of which 796 genes were downregulated and 818 genes were upregulated (Fig. 4B). When these DEGs were compared, the expression of 185 genes was significantly changed at both 10 and 18 DAP, while the expression of 1015 and 1429 genes was specifically changed 10 and 18 DAP, respectively (Fig. 4C). To further understand the function of these DEGs, GO annotation analysis was performed (Fig S4). Both 10 DAP and 18 DAP, genes in metabolic processes were the most highly represented in the biological process category (540/644 genes 10 and 18 DAP, respectively), suggesting that certain important metabolic activities during seed development were affected in the *Clbg1*-mutant lines; genes associated with the membrane (382 genes 10 DAP/492 genes 18 DAP) were the most enriched in the cellular component category; and catalytic activity-related genes (478 genes 10 DAP and 635 genes 18 DAP) were prominently represented in the category of molecular function (Fig S4A, B). Moreover, a GO enrichment analysis was also conducted for these DEGs (Fig. 5). The top 16 enriched GO terms 10 and 18 DAP are shown in Fig. 5A, B, respectively. Ten days after pollination, “biological regulation”, “regulation of metabolic process”, “regulation of macromolecule metabolic process”, “regulation of cellular metabolic process” and “regulation of nitrogen compound metabolic process” were the top five GO terms associated with gene enrichment, in descending order. Eighteen days after pollination, “microtubule-based process”, “cytoskeletal protein binding”, “microtubule binding”, “tubulin binding” and “cell cycle process” were the GO terms ranked among the top five with the most genes. Tubulin family proteins are the main constituents of microtubules in living cells, and microtubules are among the vital components that make up the cytoskeleton. Overall, cytoskeleton-related genes were enriched in the DEGs in the *Clbg1*-mutant line 18 DAP. In addition, genes involved in mitotic cell cycle processes and motor activity were also highly enriched. Taken together, the GO analysis results lay the foundation for further investigating specific pathways that are affected in the *Clbg1*-mutant lines during seed development and ultimately lead to smaller seeds.

**Fig. 6:**
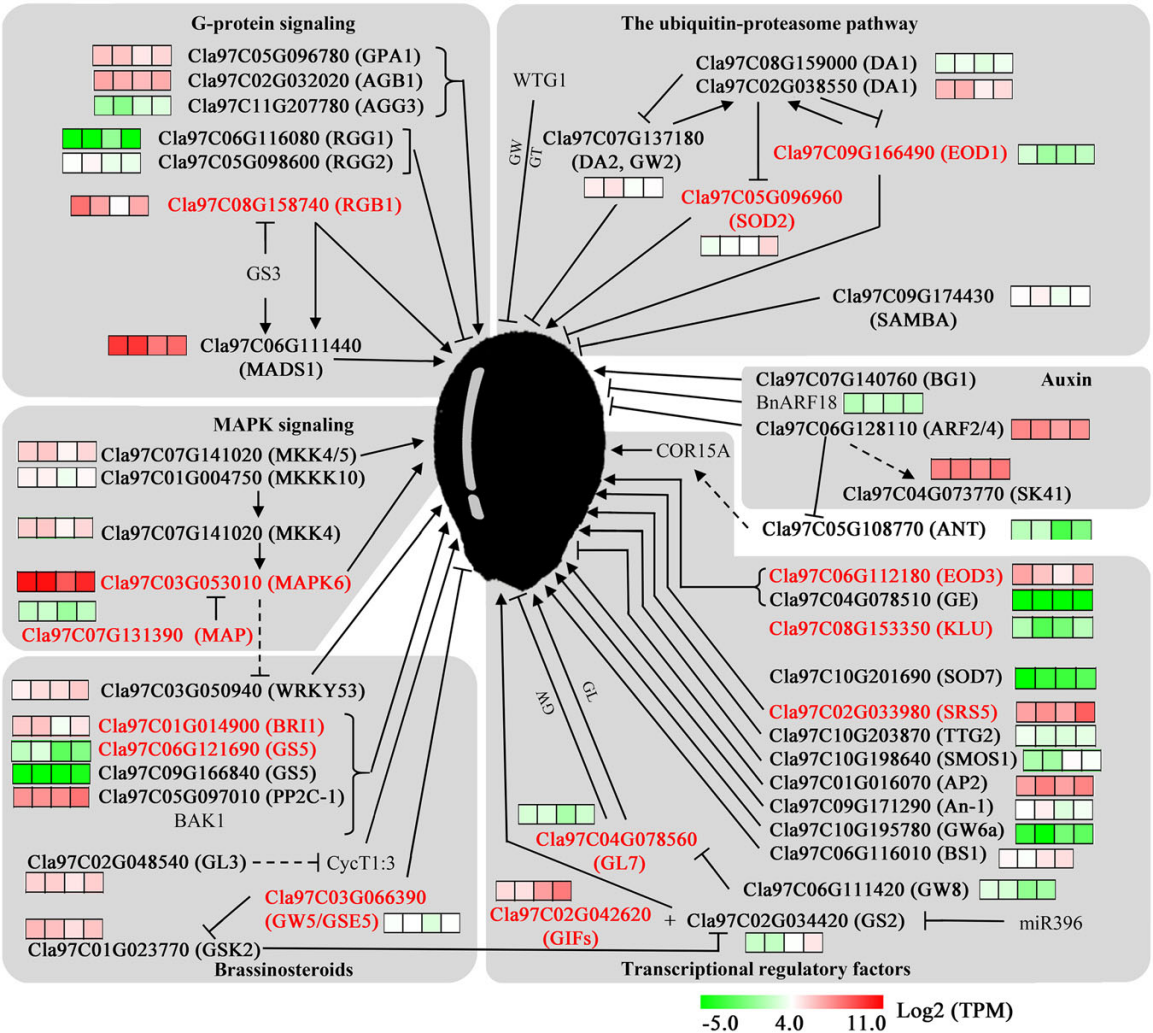
The major signaling pathways of seed size control according to Li et al.^7^. Dashed lines represent unclear genetic relationships. Seed size regulators in *Arabidopsis*, rice, and other species are shown in parentheses. The heat map near each gene shows the log2 (TPM) values in the *Clbg1*-L6 line and the WT line 10 and 18 DAP. The genes that were probably involved in watermelon seed size control are marked in red

In addition, to gain more insight into seed size variation in the *Clbg1*-mutant lines, the expression of homologous watermelon genes in the recently identified signaling pathways that control seed size was also analyzed in both the *Clbg1*-mutant and WT lines 10 and 18 DAP. In the ubiquitin-proteasome pathway, only the expression of Cla97C09G166490 (*EOD1*) and Cla97C05G096960 (*SOD2*) was significantly changed in the *Clbg1-*L6 mutant line, compared to that in the WT line, as shown in Fig. 6. The expression of Cla97C09G166490 (*EOD1*) was significantly higher in the *Clbg1-*L6 mutant line than in the WT line 10 DAP, consistent with its negative role in regulating seed size in Arabidopsis^14^. In addition, Cla97C05G096960 (*SOD2*) was significantly downregulated in the *Clbg1-*L6 mutant line 18 DAP, which may contribute to the small seed size. In G protein signaling, the expression levels of Cla97C11G207780 (*AGG3*) and Cla97C06G116080 (*RGG1*) were relatively low in both the *Clbg1-*L6 mutant and WT lines. Among the four highly expressed genes Cla97C05G096780 (*GPA1*), Cla97C02G032020 (*AGB1*), Cla97C08G158740 (*RGB1*) and Cla97C06G111440 (MADS1), only Cla97C08G158740 (*RGB1*) was differentially expressed between the *Clbg1*-mutant and WT lines. Cla97C08G158740 (*RGB1*) had significantly low expression in the *Clbg1*-mutant line 18 DAP. Based on its positive effect on seed size regulation, low Cla97C08G158740 expression may lead to smaller seed size. In the mitogen-activated protein kinase (*MAPK*) signaling pathway, Cla97C07G141020 (*MKK4/5*) and Cla97C01G004750 (*MKKK10*) showed no differential expression between the *Clbg1-*L6 mutant and WT lines. Cla97C03G053010 (*MAPK6*) was significantly downregulated in the *Clbg1-*L6 mutant line, which may have resulted in a smaller seed size. Although Cla97C07G131390 (*MAP*) was differentially expressed between the *Clbg1-*L6 mutant and WT lines 18 DAP, it may be less important due to its relatively low expression. Three genes in the auxin pathway, namely, Cla97C07G140760 (*BG1*), Cla97C06G128110 (*ARF2/4*), and Cla97C04G073770 (*SK41*), showed no differential expression between the *Clbg1-*L6 mutant and WT lines. In the brassinosteroid pathway, only Cla97C01G014900 (*BRI1*), Cla97C06G121690 (*GS5*), and Cla97C03G066390 (*GW5/GSE5*) were differentially expressed between the *Clbg1-*L6 mutant and WT lines. The expression of Cla97C01G014900 (*BRI1*) was significantly decreased in the *Clbg1-*L6 mutant line 18 DAP, indicating that it may play a role in reducing seed size. Cla97C06G121690 (*GS5*) was significantly downregulated in the *Clbg1-*L6 mutant line compared to that in the WT line; however, its role in regulating seed size at the transcriptional level may be less profound because of its relatively low expression. Moreover, the expression of the negative seed size regulator *GW5/GSE5* (Cla97C03G066390) was downregulated in the *Clbg1-*L6 mutant line 18 DAP, which is inconsistent with its role in regulating seed size in rice^36^. Among the 15 transcriptional regulatory factors, Cla97C06G112180 (*EOD3*), Cla97C08G153350 (*KLU*), Cla97C02G033980 (*SRS5*), Cla97C04G078560 (*GL7*), and Cla97C02G042620 (*GIF*) were differentially expressed between the *Clbg1-*L6 mutant and WT lines, and all of these genes were downregulated in the *Clbg1-*L6 mutant line 18 DAP, consistent with their positive role in regulating seed size.

### *Clbg1* mutation promoted seed germination and altered the expression of associated genes

**Fig. 7:**
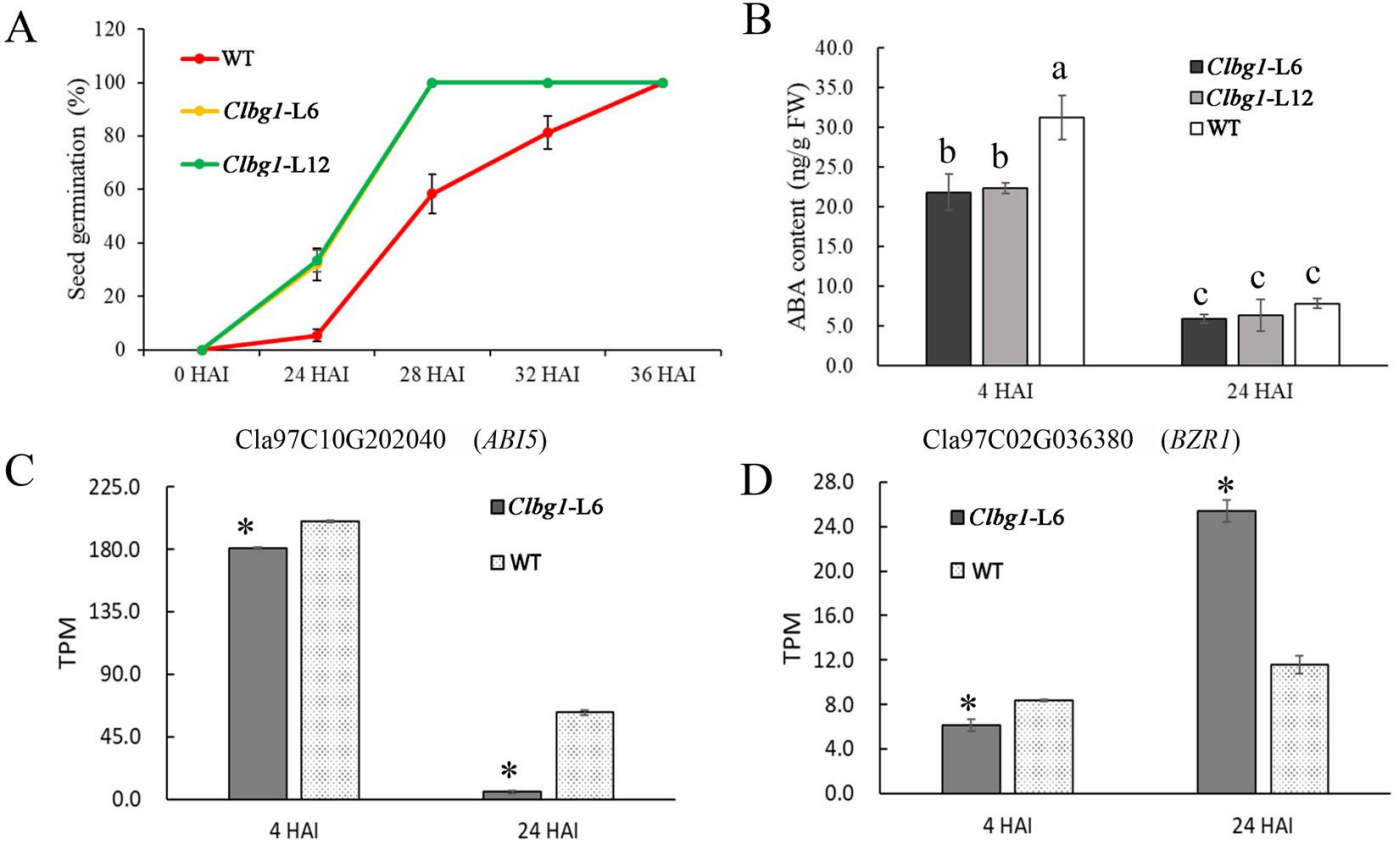
Variations in seed germination rate, ABA content, and gene expression after seed imbibition. **A** Variation in seed germination rate in the *Clbg1*-L6, *Clbg1*-L12, and wild-type lines. **B** Variation in ABA content in the *Clbg1*-L6, *Clbg1*-L12 and wild-type lines 4 and 24 HAI. **C**, **D** Expression of the *ABI5* and *BZR1* in *Clbg1*-L6 and WT lines 4 and 24 HAI. Standard error is indicated. Different letters represent significant differences as determined using ANOVA followed by Tukey’s HSD test (*p* < 0.05). * Indicates expression values that are significantly different at the 0.05 level compared to those of the WT line

To investigate the effect of *ClBG1* on seed germination, the germination rate was recorded periodically after seed imbibition. Both the *Clbg1-*L6 and *Clbg1-*L12 mutant lines exhibited very similar germination potential, which was higher than that in WT during the germination process (Fig. 7A). Twenty-four hours after imbibition (HAI), both the *Clbg1-*L6 and *Clbg1-*L12 mutant lines reached a germination rate of approximately 33.3%, while this rate was only 5.3% in the WT line. The *Clbg1*-mutant lines had completely germinated 28 HAI; however, it took another 8 hours for the WT seeds to completely germinate. The ABA content variation was significantly decreased 24 HAI compared to that at 4 HAI in both the *Clbg1*-mutant and the WT lines. Moreover, there was an obvious decrease in ABA content in the *Clbg1*-mutant lines compared to that in WT 4 HAI (Fig. 7B). Therefore, seed germination occurred earlier and the germination rate was higher in the *Clbg1*-mutant lines. Twenty-four hours after imbibition, the ABA content was similar in the *Clbg1*-mutant and WT lines, which was in accordance with the similar seed germination rate 24 HAI (Fig. 7A, B). Although the seed germination potential of the *Clbg1*-mutant lines was increased, radicle elongation seemed to be have been delayed (Fig S5).

**Fig. 8:**
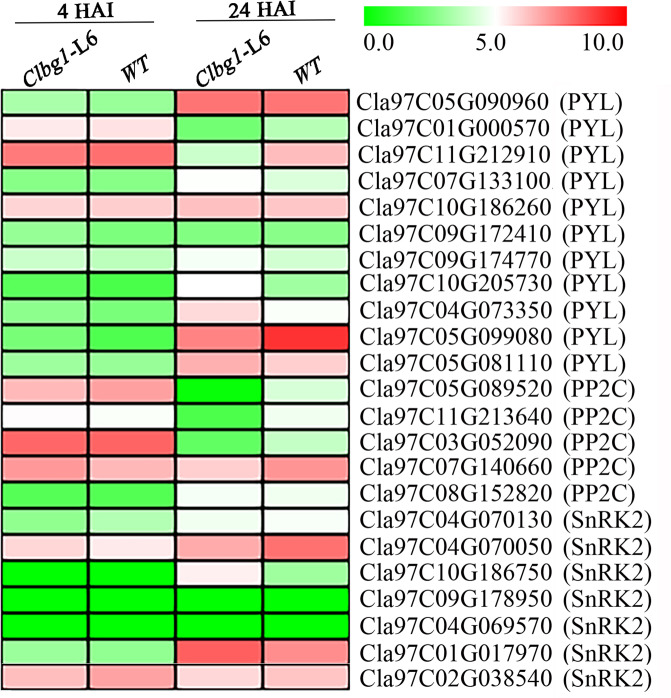
Expression of ABA signaling genes in the *Clbg1*-L6 and WT lines 4 and 24 HAI. The expression values of each gene are represented as log2 (TPM)

To clarify the increased seed germination potential in *Clbg1*-mutant lines at the transcriptional level, the expression of certain seed germination indicator genes and ABA signaling pathway genes was analyzed. The expression of seed germination repressors, such as Cla97 C10G202040 (*ABI5*), was significantly decreased in the *Clbg1-*L6 mutant line compared to the WT line at both 4 and 24 HAI (Fig. 7C). The expression of Cla97C02G036380 (*BZR1*), which negatively mediates ABA signaling to promote seed germination, was significantly increased 24 HAI in the *Clbg1-*L6 mutant line (Fig. 7D). In the ABA signaling pathway, there were 11 putative *ClPYL* genes, 5 putative group A *ClPP2C* genes, and 8 putative subclass III *ClSnRK2* genes (Fig. 8). Among the *ClPYL* genes, most were not expressed significantly differently in the *Clbg1-*L6 mutant and WT lines. The expression of Cla97C01G000570 (*PYL*), Cla97C11G212910 (*PYL*), and Cla97C05G099080 (*PYL*) was significantly downregulated in the *Clbg1-*L6 mutant line 24 HAI, while Cla97C10G205730 (*PYL*) was significantly upregulated. Among the five group A *ClPP2C* genes, the expression of four genes, namely, Cla97C05G089520, Cla97C11G213640, Cla97C03G052090, and Cla97C07G140660, but not that of Cla97C08G152820 (*PP2C*), was significantly decreased in the *Clbg1-*L6 mutant line compared the WT line. As for the *ClSnRK2* genes, Cla97C09G178950 and Cla97C04G069570 were not expressed in any of the samples examined. Except for Cla97C04G070050 and Cla97C10G186750, which showed significant expression differences between the WT and *Clbg1-*L6 mutant lines, *ClSnRK2* genes were not differentially expressed.

## Discussion

ABA, a classic multifaceted phytohormone, regulates many life processes of plants, including seed germination and dormancy, fruit development and ripening, and stress adaption^37^. During these processes, ABA function depends on the variation of its level, which is mainly controlled by de novo biosynthesis, hydroxylation, and conjugation. The hydrolysis of ABA-glucose ester mediated by β–glucosidase, which releases active ABA, is thought to be faster in increasing ABA levels than the de novo biosynthesis pathway^33^ and is considered to play a key role in the regulation of endogenous ABA content in response to abiotic stresses and thus influences fruit physiology^38–40^. The mechanism of ABA glucosylation effects on ABA concentration, which influences seed morphology, has been rarely addressed. In this study, we found that *ClBG1* (Cla97C08G153160) was highly expressed during cultivated watermelon ripening but expressed at low levels in nonripening wild watermelon PI296341-FR (Fig. 1B), suggesting that this gene may be involved in ABA-mediated watermelon fruit ripening. The development of various genome-editing systems has greatly facilitated studies on specific gene functions because they can be used to disrupt genes via frameshift mutations, including the creation of premature stop codons, and the production of different proteins^41^. Taking advantage of the CRISPR/Cas9 system, we abrogated the function of *ClBG1* in watermelon; however, no obvious changes in the fruit ripening phenotype were observed, which may be due to the redundancy of gene family members. In an unexpected finding, the seed size was significantly decreased in the *Clbg1*-mutant lines and seed germination was promoted due to decreased ABA content (Figs. 2, 3D, and 7A). As shown in Fig S6, no significant differences were found between the WT and *Clbg1*-mutant lines in the expression of *ClNCED* or *ClCYP707A* genes during seed development. These results suggest that the decrease in ABA content in *Clbg1*-mutated seeds was not caused by the de novo biosynthesis or degradation of ABA but by the dysfunction of *ClBG1* due to the mutation, thereby decreasing watermelon seed size and promoting seed germination.

Seed size is an important agronomic trait in watermelon breeding. Several studies have identified QTLs related to seed size and weight in watermelon, mainly throughout chromosomes 1, 2, 3, 5, 6, 8, and 11 (refs. ^42–45^). In this study, we found that dysfunction of *ClBG1* led to decreased seed size and weight. Notably, *ClBG1*, located on chromosome 8 (21432735-21439034, watermelon 97103 V2), was physically near to the seed size QTL ClSS8.2 identified by Ren et al.^43^ and Prothro et al.^42^. This finding confirms that *ClBG1* may play a role in watermelon seed size control. In addition, the shape and size of plant organs are controlled by programmed cell division and cell expansion. Specifically, the spatiotemporal patterns of cell division and cell enlargement are mainly regulated by the reorientation of microtubule arrays^46,47^. In this study, the GO term enrichment analysis of the DEGs revealed that microtubule-, tubulin- and cell cycle-related genes were strongly affected by mutation in the *Clbg1* gene, which was consistent with the alteration of cell number and size (Figs. 3, 5). Although the cell size was significantly increased, seed size and weight were decreased in the *Clbg1-*mutant lines, which indicates that cell number is the most important factor in seed size determination. In the major signaling pathways of seed size control, the function of some genes is conserved among different species. For example, in the MAPK signaling pathway, the OsMKKK10-OsMKK4-OsMAPK6 cascade controls rice grain size, in which OsMKK4 and OsMAPK6 can be sequentially phosphorylated and activated by OsMKKK10, and the expression of OsMAPK6 is positively associated with cell proliferation in the spikelet hull^20^. In watermelon, the expression of Cla97C01G004750 (*MKKK10*), Cla97C07G141020 (*MKK4*), and Cla97C03G053010 (*MAPK6*) was also positively correlated with seed size (Fig. 6). On the other hand, among the brassinosteroid-related genes, the downregulation of *GSE5/GW5* in rice led to large grains because of extensive cell proliferation in the spikelet hull^36^. However, downregulation of these genes had the opposite effect in watermelon. The expression of Cla97C03G066390 (*GSE5/GW5*) was low in the *Clbg1*-mutant lines with small seeds. Therefore, it can be presumed that GSE5/GW5 in rice and watermelon might have different cofactors, which leads to the opposite effects on seed size. Further investigations are needed to discover the different roles of genes in seed size control in different species.

## Materials and methods

### Plant materials

The watermelon inbred line ZXJM was used in the transformation experiments mediated by *Agrobacterium tumefaciens* strain GV3101. ZXJM is a cultivated watermelon with red flesh and medium seed size. PI296341-FR is a nonripening wild-type watermelon variety that has white flesh and medium seed size. Flowers were artificially pollinated and labeled on the day of flowering. PI296341-FR, *ClBG1*-edited and wild-type (WT) plants (plants derived from transformation but with no genome editing and free of Cas9) were grown under standard greenhouse conditions in the Beijing Vegetable Research Center (BVRC). The seeds were periodically harvested.

### Phylogenetic analysis of BGs

The amino acid sequences of the ClBG proteins were aligned with the relatively well-studied BGs in other species, such as SlBG2 in tomato, VvBG1 in grape, AtBG1 and AtBG2 in *Arabidopsis,* and FaBG3 in strawberry, using ClustalX 2.0.12 software with default settings. Phylogenetic trees were constructed with MEGA 4.0.2 software using the neighbor-joining (N-J) method, and the reliability of the different phylogenetic groups was evaluated by bootstrap analysis with 1000 replicates. Tree files were viewed and edited with MEGA 4.0.2 software.

### Vector construction and Agrobacterium-mediated transformation in watermelon

The binary CRISPR/Cas9 vector pBSE401 was provided by Q.J. Chen at China Agricultural University, and the vector was constructed as previously described by Xing et al.^48^. Specific single guide RNAs (sgRNAs) targeted to *ClBG1* were selected according to the assessment with CRISPR-P (http://cbi.hzau.edu.cn/crispr/). The target sequence cloned into the pBSE401 vector was named pBSE401-*ClBG1*, which was used to transform the watermelon cultivar ZXJM by the Agrobacterium-mediated transformation method as previously described^49^. The transgenic watermelon lines were selected based on basta resistance. The primers used for vector construction are listed in Supplementary Table S1.

### Genomic DNA extraction and mutation detection

Genomic DNA was extracted from young leaves of T0-T4 transgenic plants, which was then used for creating templates to amplify the specific fragments in the ClBG1 gene using primers flanking two targeted sites (Supplementary Table S1). PCR was conducted under the following conditions: 94 °C/5 min; 94 °C/30 s, 56 °C/30 s, and 72 °C/1 min (35 cycles); and 72 °C/10 min as the final extension. PCR products were directly sequenced using the Sanger method by Tianyi Huiyuan Biotech Company (Beijing, China). The transgenic plants were also verified as Cas9-free with primers specific for Cas9 (Supplementary Table S1). PCR was conducted under the following conditions: 94 °C/5 min; 94 °C/30 s, 60 °C/30 s, and 72 °C/1 min (29 cycles); and 72 °C/10 min as the final extension.

### Measurement of seed size and weight

Seeds from fully mature watermelons were collected and dried. Seeds from each watermelon were randomly selected and photographed. Maximum seed length and width were measured with ImageJ software (http://rsbweb.nih.gov/ij/). Seed weight was measured using an electronic balance. The seed weight data of 6 watermelons were combined according to plant line. This seed weight is expressed as the average of these 6 measurements for each watermelon line.

### Histological analysis of mature seeds

Mature seeds of the wild-type and *Clbg1*-mutant lines were collected. Paraffin-embedded sections of these mature seeds were obtained by cutting in the longitudinal direction according to the method described by Dai et al.^50^. Images were obtained using an automatic digital pathology slide scanner (KF-PRO-120, Jiangfeng Biological Information Technology Co., Ltd, Ningbo, China) and viewed with software K-Viewer (http://www.kfbio.cn/download.php). Cells were counted in areas of the same size (40,000 μm²), as indicated in the boxes in Fig. 3, and the boxed area divided by cell number was the cell size. For wild-type and ClBG1-mutant line 6, at least three seeds were sectioned, and three sections of each seed were analyzed.

### Seed germination experiments

Watermelon seeds of the wild-type and *ClBG1*-mutant lines with uniform plumpness were first soaked in sterilized water for 4 hours in a beaker, and then some seeds were collected as samples 4 hours after imbibition (HAI). The remaining seeds were placed into petri dishes, which were lined with moist seed-germinating paper. The germination rate (number of germinated seeds/number of total seeds) was recorded 24, 28, 32, and 36 HAI. At least 100 seeds were used for each genotype. The seed samples were immediately collected and frozen in liquid nitrogen 4 HAI and 24 HAI and stored at −80 °C until use.

### Determination of ABA content

Determination of ABA content was performed using a ESI-HPLC-MS system according to the method described in our previous study^35^. The ABA standard ((±)-abscisic acid, A1049, Sigma, St Louis, MO, USA) was used to determine the retention time and mass spectrometric information of ABA. Three biological replicates of each sample were analyzed.

### RNA library construction, sequencing, and analysis

RNA was isolated from lyophilized tissues with a plant RNA purification reagent kit (Invitrogen, USA). The concentration, quality, and purity of the RNA were detected with an Agilent 2100 Bioanalyzer RNA 6000 Nano kit (Agilent, USA). Twenty-four RNA libraries (seeds of the wild-type and *Clbg1*-L6 lines 10 DAP, 18 DAP, 4 HAI, and 24 HAI, each with three replicates) were generated with a TruSeqTM RNA Sample Prep kit (Illumina, USA) and sequenced on an Illumina HiSeq 4000 in paired-end 150-bp read mode. The data were analyzed free online with the Majorbio I-Sanger Cloud Platform (www.i-sanger.com).

## Supplementary information

Figure S1

Figure S2

Figure S3

Figure S4

Figure S5

Figure S6

Table S1
