# Supplementary figures and images for: CRISPR/Cas9-mediated mutagenesis of ClBG1 decreased seed size and promoted seed germination in watermelon
Source: Hortic Res. 2021 Apr 1;8:70. doi: 10.1038/s41438-021-00506-1 (PMC8012358; doi:10.1038/s41438-021-00506-1)

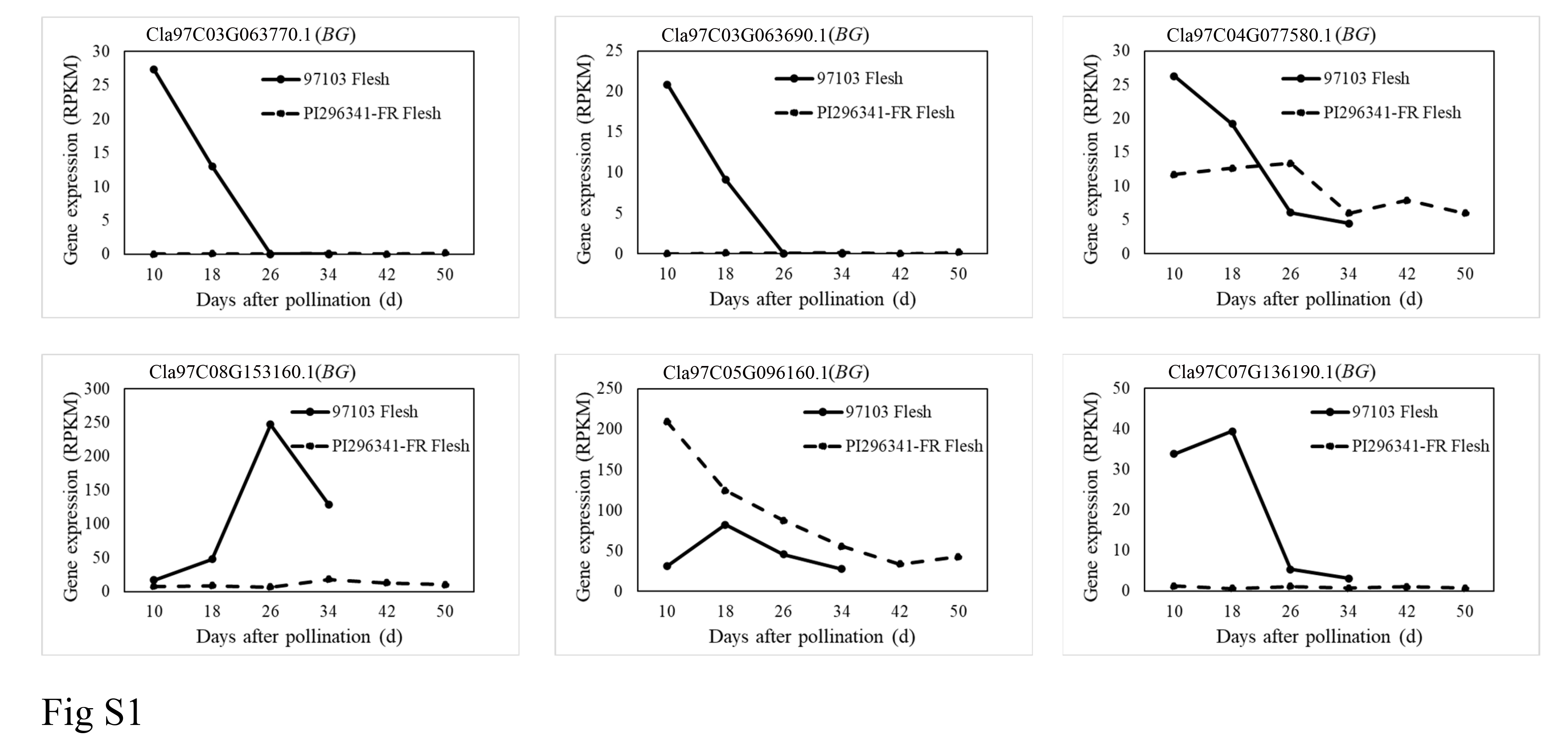

Supplement: Supplementary file 1 — Figure S1 [file 41438_2021_506_MOESM1_ESM.jpg]

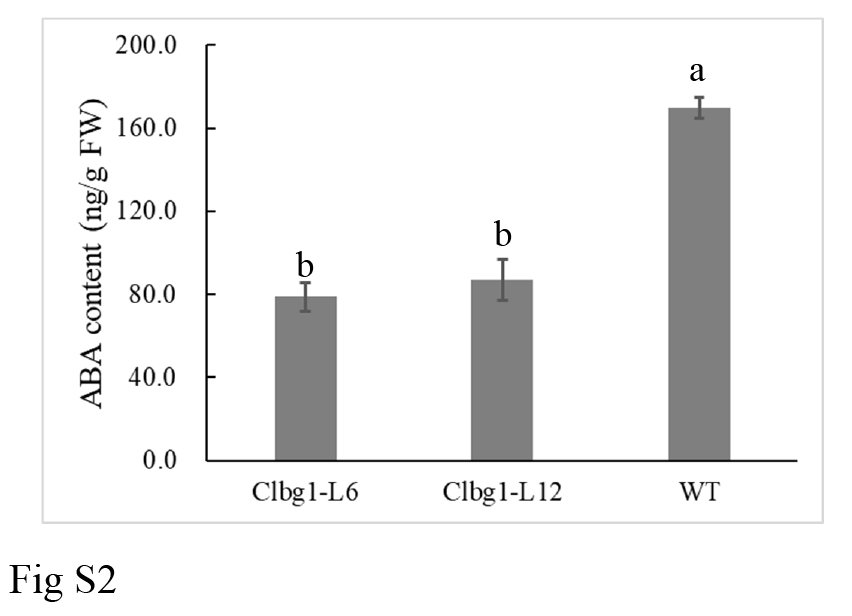

Supplement: Supplementary file 2 — Figure S2 [file 41438_2021_506_MOESM2_ESM.jpg]

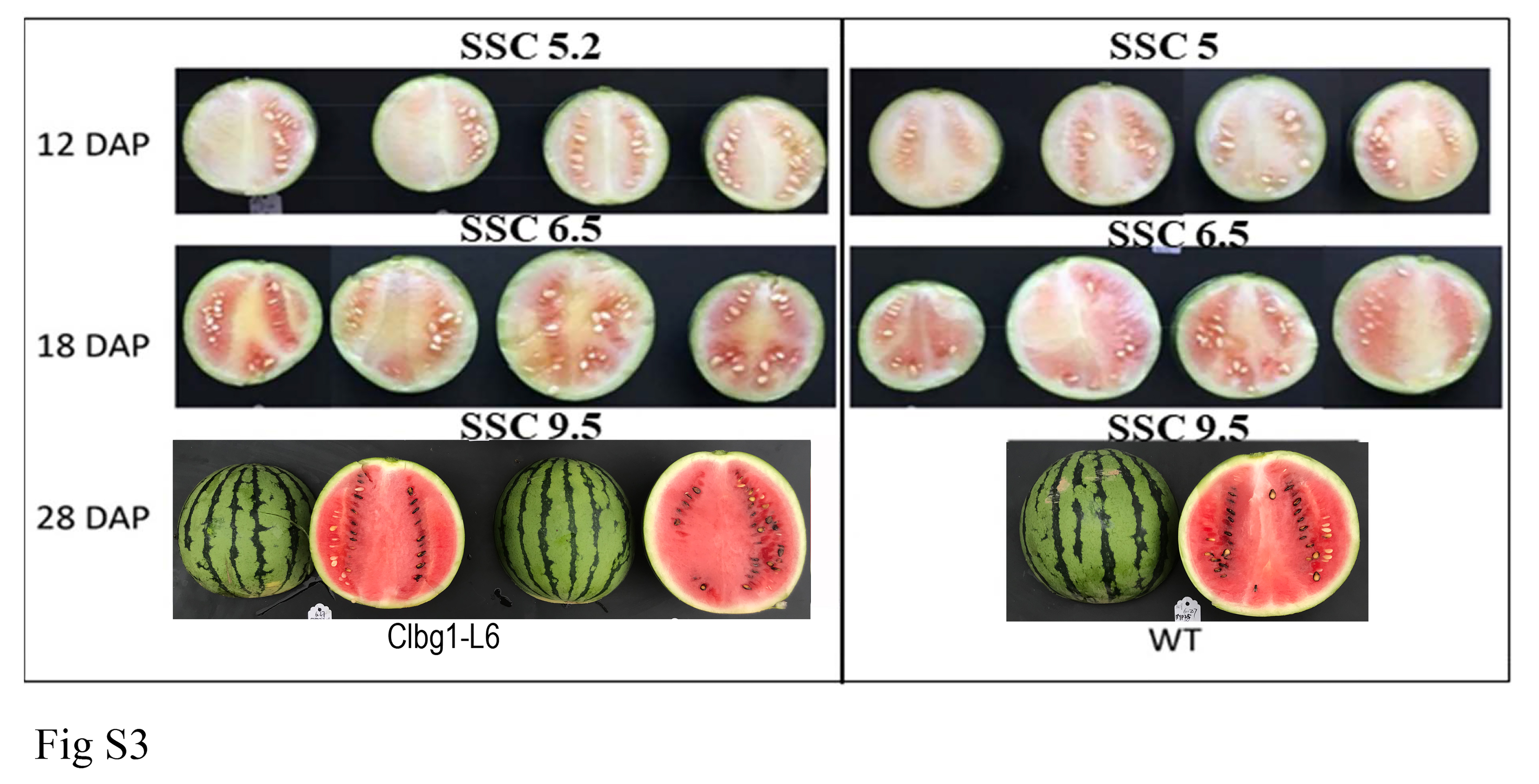

Supplement: Supplementary file 3 — Figure S3 [file 41438_2021_506_MOESM3_ESM.jpg]

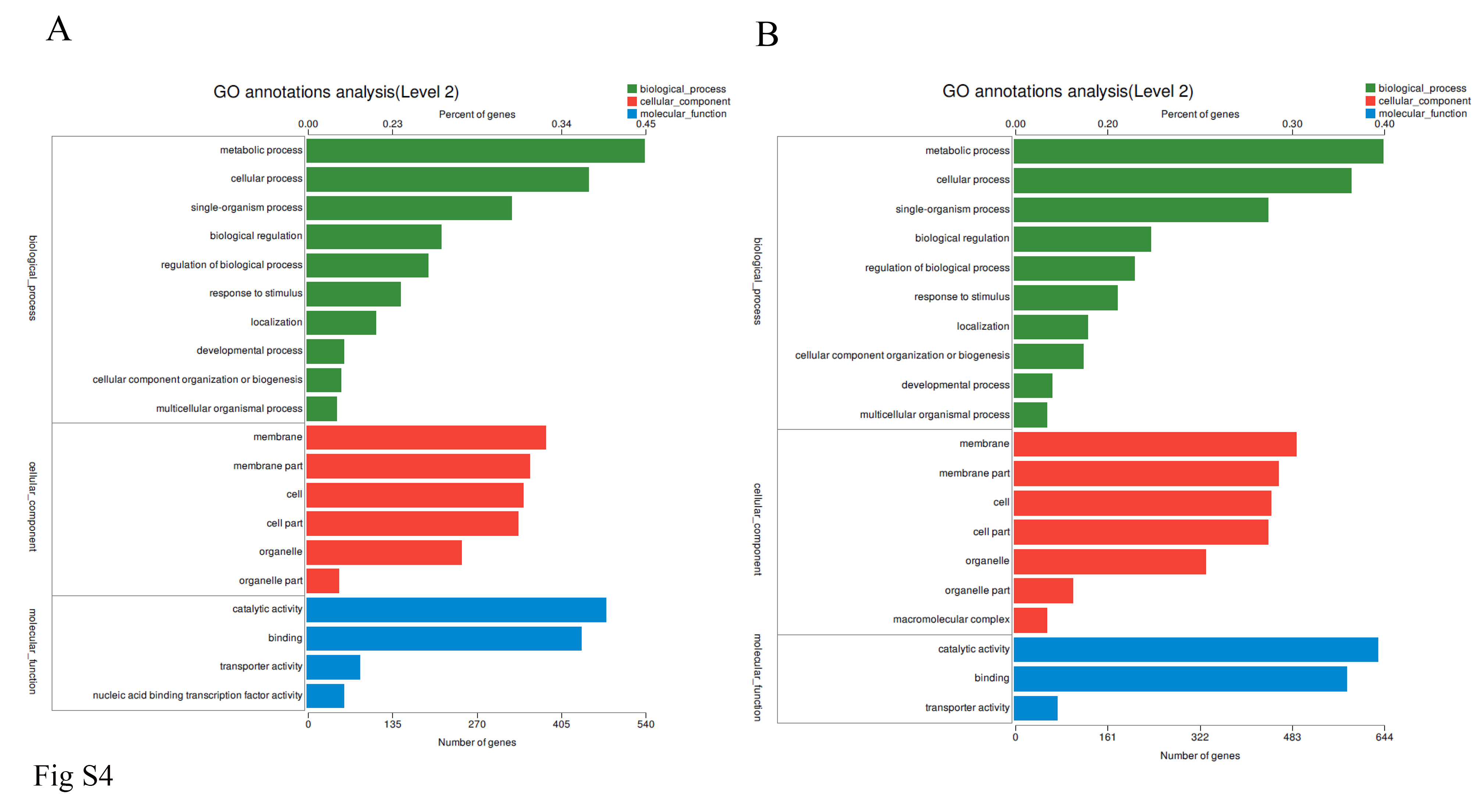

Supplement: Supplementary file 4 — Figure S4 [file 41438_2021_506_MOESM4_ESM.jpg]

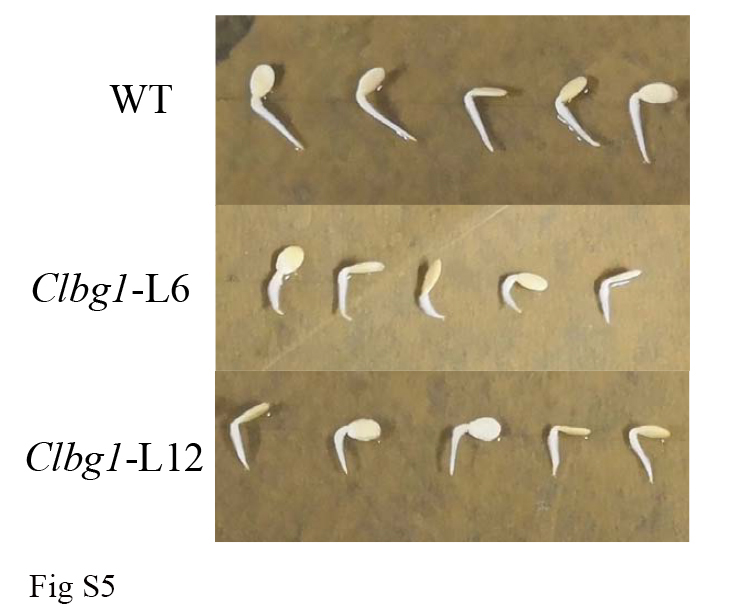

Supplement: Supplementary file 5 — Figure S5 [file 41438_2021_506_MOESM5_ESM.jpg]

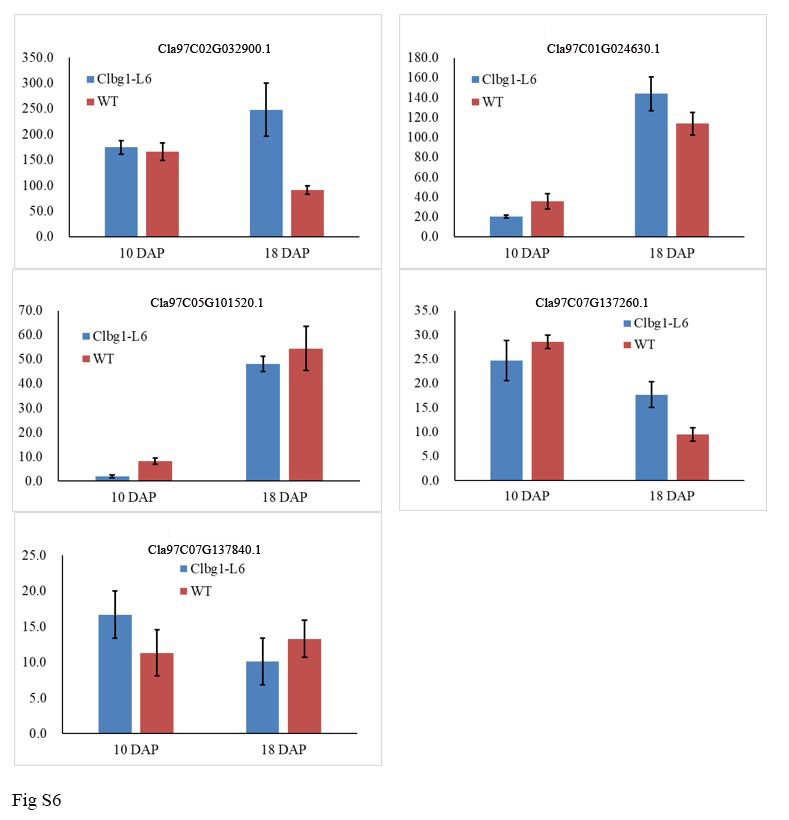

Supplement: Supplementary file 6 — Figure S6 [file 41438_2021_506_MOESM6_ESM.jpg]
