# Supplementary material for: CRISPR/Cas9-mediated mutagenesis of ClBG1 decreased seed size and promoted seed germination in watermelon
Source: Hortic Res. 2021 Apr 1;8:70. doi: 10.1038/s41438-021-00506-1 (PMC8012358; doi:10.1038/s41438-021-00506-1)
Supplement: Supplementary file 7 — Table S1 [file 41438_2021_506_MOESM7_ESM.docx]

Table S1 Primers used in this study.

| Gene | Forward primer |  |
| --- | --- | --- |
| Primers for vector construction | | |
| DT1-BsF | ATATATGGTCTCGATTGGAGGAAGATTCGCCACCGTGTT |  |
| DT1-F0 | TGGAGGAAGATTCGCCACCGTGTTTTAGAGCTAGAAATAGC |  |
| DT2-R0 | AACAGCGGATTTGTTTTCGGAACAATCTCTTAGTCGACTCTAC |  |
| DT2-BsR | ATTATTGGTCTCGAAACAGCGGATTTGTTTTCGGAAC |  |
| Primers used for *ClBG1* mutation detection | | |
| BGJCF1 | TTCTCCTGAGTTGGATTTCCACATA |  |
| BGJCR | ACTACGGTTTTGATTCTCTTCATCG |  |
| Primers used for cas9 detection | | |
| 35S-F1 | CAAGACCCTTCCTCTATATAAGGA |  |
| zCas9-951R | GAGGTTATCCAGGTCATCG |  |
